# *NCKAP1L* defects lead to a novel syndrome combining immunodeficiency, lymphoproliferation, and hyperinflammation

**DOI:** 10.1084/jem.20192275

**Published:** 2020-08-06

**Authors:** Carla Noemi Castro, Michelle Rosenzwajg, Raphael Carapito, Mohammad Shahrooei, Martina Konantz, Amjad Khan, Zhichao Miao, Miriam Groß, Thibaud Tranchant, Mirjana Radosavljevic, Nicodème Paul, Tristan Stemmelen, Fabien Pitoiset, Aurélie Hirschler, Benoit Nespola, Anne Molitor, Véronique Rolli, Angélique Pichot, Laura Eva Faletti, Bruno Rinaldi, Sylvie Friant, Mark Mednikov, Hatice Karauzum, M. Javad Aman, Christine Carapito, Claudia Lengerke, Vahid Ziaee, Wafaa Eyaid, Stephan Ehl, Fayhan Alroqi, Nima Parvaneh, Seiamak Bahram

**Affiliations:** 1 Institute for Immunodeficiency, Center for Chronic Immunodeficiency, Medical Center-University of Freiburg, Faculty of Medicine, University of Freiburg, Freiburg, Germany; 2 Assistance Publique-Hôpitaux de Paris, Pitié-Salpêtrière Hospital, Biotherapy (Centre d’Investigation Clinique intégré en Biothérapies & immunologie; CIC-BTi) and Inflammation-Immunopathology-Biotherapy Department (i2B), Paris, France; 3 Sorbonne Université, Institut National de la Santé et de la Recherche Médicale UMR_S 959, Immunology-Immunopathology-Immunotherapy (i3), Paris, France; 4 Laboratoire d’ImmunoRhumatologie Moléculaire, Plateforme GENOMAX, Institut National de la Santé et de la Recherche Médicale UMR_S 1109, Faculté de Médecine, Fédération Hospitalo-Universitaire OMICARE, Fédération de Médecine Translationnelle de Strasbourg, Laboratory of Excellence TRANSPLANTEX, Université de Strasbourg, Strasbourg, France; 5 Service d’Immunologie Biologique, Plateau Technique de Biologie, Pôle de Biologie, Nouvel Hôpital Civil, Hôpitaux Universitaires de Strasbourg, Strasbourg, France; 6 Specialized Immunology Laboratory of Dr. Shahrooei, Sina Medical Complex, Ahvaz, Iran; 7 Department of Microbiology and Immunology, University of Leuven, Leuven, Belgium; 8 University of Basel and University Hospital Basel, Department of Biomedicine, Basel, Switzerland; 9 Department of Pediatrics, King Abdulaziz Medical City, King Abdullah Specialized Children's Hospital, Riyadh, Saudi Arabia; 10 King Saud bin Abdulaziz University for Health Sciences, Riyadh, Saudi Arabia; 11 King Abdullah International Medical Research Center, Riyadh, Saudi Arabia; 12 European Molecular Biology Laboratory, European Bioinformatics Institute, Wellcome Genome Campus, Hinxton, Cambridge, UK; 13 Shanghai Fourth People's Hospital Affiliated to Tongji University School of Medicine, Shanghai, Hongkou, China; 14 Laboratoire de Spectrométrie de Masse Bio-Organique, Institut Pluridisciplinaire Hubert Curien, UMR 7178, Université de Strasbourg, Centre National de la Recherche Scientifique, Strasbourg, France; 15 Department of Pediatrics and Adolescent Medicine, Medical Center-University of Freiburg, Faculty of Medicine, University of Freiburg, Freiburg, Germany; 16 Laboratoire de Génétique Moléculaire, Génomique, Microbiologie, UMR7156/Université de Strasbourg, Centre National de la Recherche Scientifique, Strasbourg, France; 17 Integrated BioTherapeutics, Inc., Rockville, MD; 18 Pediatric Rheumatology Research Group, Rheumatology Research Center, Tehran University of Medical Sciences, Tehran, Iran; 19 Department of Pediatrics, Tehran University of Medical Sciences, Tehran, Iran; 20 Division of Allergy and Clinical Immunology, Department of Pediatrics, Tehran University of Medical Sciences, Tehran, Iran; 21 Research Center for Immunodeficiencies, Tehran University of Medical Sciences, Tehran, Iran

## Abstract

The Nck-associated protein 1–like (*NCKAP1L*) gene, alternatively called hematopoietic protein 1 (*HEM-1*), encodes a hematopoietic lineage–specific regulator of the actin cytoskeleton. *Nckap1l*-deficient mice have anomalies in lymphocyte development, phagocytosis, and neutrophil migration. Here we report, for the first time, *NCKAP1L* deficiency cases in humans. In two unrelated patients of Middle Eastern origin, recessive mutations in *NCKAP1L* abolishing protein expression led to immunodeficiency, lymphoproliferation, and hyperinflammation with features of hemophagocytic lymphohistiocytosis. Immunophenotyping showed an inverted CD4/CD8 ratio with a major shift of both CD4^+^ and CD8^+^ cells toward memory compartments, in line with combined RNA-seq/proteomics analyses revealing a T cell exhaustion signature. Consistent with the core function of NCKAP1L in the reorganization of the actin cytoskeleton, patients’ T cells displayed impaired early activation, immune synapse morphology, and leading edge formation. Moreover, knockdown of *nckap1l* in zebrafish led to defects in neutrophil migration. Hence, *NCKAP1L* mutations lead to broad immune dysregulation in humans, which could be classified within actinopathies.

## Teaser

Biallelic mutations in *NCKAP1L*, a regulator of the actin cytoskeleton, cause immunodeficiency, lymphoproliferation, and hyperinflammation with features of hemophagocytic lymphohistiocytosis. Impaired immune synapse formation, early T cell activation and leading edge formation, and defective neutrophil migration characterize this novel “actinopathy.”

## Introduction

Circumscription of the innate or adaptive immune response is equally important to its initiation, as an otherwise unhinged immune response would result in overt pathology, including lymphoproliferation, autoimmunity, hyperinflammation, and/or immunodeficiency (Delmonte et al., 2019). Most of these manifestations are part of hemophagocytic lymphohistiocytosis (HLH), a life-threatening disease associated with uncontrolled T cells, natural killer (NK) cells, and/or macrophage activation and excessive inflammatory cytokine secretion (Al-Samkari and Berliner, 2018). Clinically, HLH is characterized by a combination of mainly unspecific symptoms due to lymphoproliferation (e.g., splenomegaly), inflammation (e.g., fever), various system/organ dysfunctions (liver injury, central nervous system inflammation), and a number of nonpathognomonic biological abnormalities (including pancytopenia, coagulopathy, hyperlipidemia, hyperferritinemia, and sCD25 elevation). HLH is traditionally divided into two categories: primary (or familial), associated with genetic defects in lymphocyte cytotoxicity, and secondary, in which patients do not carry a mutation in genes known to predispose to HLH (Janka, 2012; Tesi and Bryceson, 2018). Secondary HLH can be triggered by a viral infection or an autoimmune or malignant disease (Al-Samkari and Berliner, 2018; Tangye et al., 2017). However, this distinction is becoming blurred, since an increasing number of inborn errors of immunity have been shown to predispose to HLH in the absence of cytotoxicity defects (Bode et al., 2015; Canna et al., 2014; Gayden et al., 2018; Lam et al., 2019).

Here we report two unrelated patients presenting with symptoms of immunodeficiency, lymphoproliferation, and inflammation, collectively defining a novel nosological entity—i.e., familial hyperinflammatory immunodeficiency with features of HLH. Unlike familial HLH, where mutations lead to defects in transport, exocytosis, or the content of cytotoxic granules in T and NK cells, the disease described here is due to homozygous mutations in Nck-associated protein 1–like (*NCKAP1L*), a key component of the actin cytoskeleton machinery. *NCKAP1L,* alternatively called hematopoietic protein-1 (*HEM-1*), is a hematopoietic lineage–restricted member of the Nap1l subunit of the WAVE (WASP-family verprolin-homologous protein) complex. It signals downstream of activated Rac to stimulate F-actin polymerization in response to engagement of various immune receptors (B cell receptor, TCR, TLR, or cytokine receptors). *Nckap1l*-deficient mice further established that the molecule is critical for activation, migration, and cellular contact formation of lymphoid and myeloid cells, including immunological synapse formation in effector cells (Park et al., 2008), which could explain the combination of impaired control of infection and continuous immune cell stimulation with inflammatory consequences.

## Results and discussion

### Clinical phenotype

**Figure 1.**
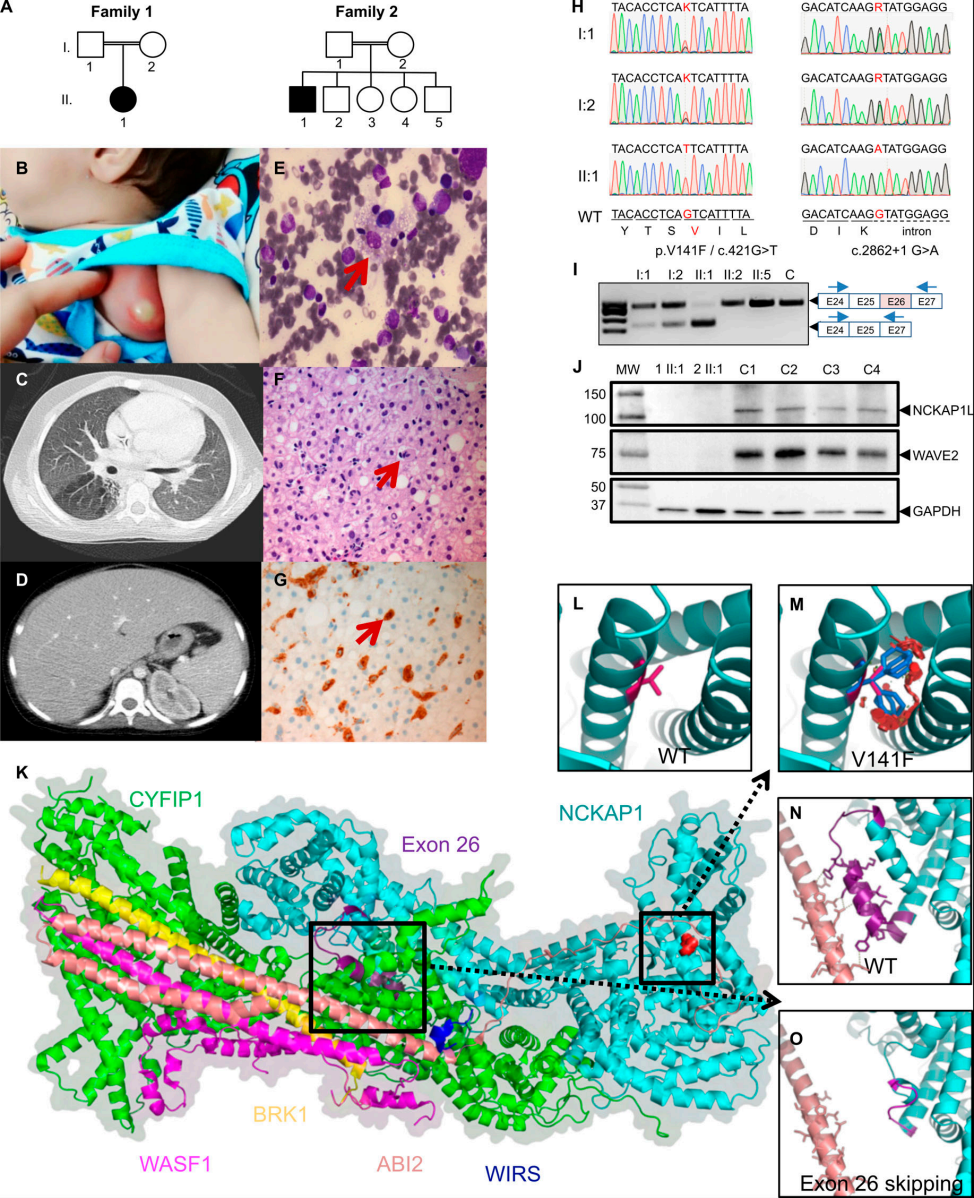
**Homozygous *NCKAP1L* mutations identified in the two families.** **(A)** Pedigrees of affected consanguineous families of Iranian (family 1) and Saudi Arabian (family 2) origins. Generations are designated by Roman and subjects by Arabic numerals. Double lines connecting parents indicate consanguinity. Squares, male subjects; circles, female subjects; filled (black) symbols indicate patients, while unfilled (white) symbols indicate unaffected family members. All family members in family 1 and all in family 2, with the exception of II.3 and II.4, were subjected to whole-exome sequencing. **(B)** Photograph of axillary lymphadenitis in patient 1. **(C–G)** In patient 2, a thoracoabdominal computed tomography scan showed right lung focal oligemia and brocheactesis (C) and massive hepatosplenomegaly (D). In the same patient, bone marrow aspirates showed hemophagocytosis (E), whereas liver staining with hematoxylin and eosin (F) or CD68 (G) showed sinusoidal dilatation with hemophagocytic histiocytosis. **(H)** Sanger traces of the disease-associated *NCKAP1L* mutations confirming autosomal recessive inheritance. Electropherograms show single base pair substitutions c.421G>T of *NCKAP1L* (NM_005337) causing V141F missense in family 1 (left panel) and a single base pair substitution c.2821+1 G>A disrupting the donor splice site of *NCKAP1L* exon 26 in family 2. **(I)** RT-PCR in PBMCs in family 2 shows homozygous skipping of exon 26 in patient II:1. **(J)** Western blot analysis of NCKAP1L complex proteins expression in PBMCs from patients and controls. NCKAP1L and WAVE2 proteins are absent in both patients. GAPDH was used as a loading control. Molecular weight (MW) markers are shown on the left in kilodaltons. **(K–O)** Structure modeling of NCKAP1L and its mutants in the context of the WAVE complex. The cartoon representation of the structure model of the NCKAP1L protein was established using NCKAP’s structure (PDB 4n78 chain B) as a template. The N- to -C is shown as a rainbow color from blue to red. NCKAP1L is shown in cyan, and the product of exon 26 (purple), V141F mutation (red), and WIRS (blue) are in close contact with ABI2 (light salmon; K). Zooming to the V141F mutation shows the WT valine as hot pink (L). The residue has close contacts with the other two helices. When mutated to phenylalanine (patient 1), the large side-chain groups result in severe atomic clash, which indicates a reorganization of the helical structure and may have an effect on ABI2 binding (M). Exon 26’s product (purple) forms close contacts (shown as yellow dashed lines) with ABI2 (N). When exon skipping occurs (patient 2), the helix in the C terminus of the exon 26 product changes to a loop region (shown in magenta; O). The contacts with ABI2 disappear in this situation.

Patient 1 is a 15-mo-old girl, the only child of healthy consanguineous parents of Iranian origin (Fig. 1 A). She received a standard set of vaccinations (tetanus, oral polio, Bacillus Calmette–Guérin [BCG] and hepatitis B) at birth. At 1.5 mo of age, she presented with fever and massive splenomegaly. Blood counts revealed a hyperleukocytosis (56,000–85,500 cells/µl) with relative lymphocytosis (3,000–51,000 cells/µl) and monocytosis (5,600–7,700 cells/µl; Table 1). She had mild anemia (hemoglobin, 9.5–10.2 g/dl) with discrete evidence for anisopoikilocytosis. A bone marrow aspirate showed normal cellularity with no visible sign of hemophagocytosis. Further immunological workup showed relative CD4 lymphocytopenia, increased serum levels of IgG (2,581 mg/dl), IgA (130 mg/dl), IgM (>1,000 mg/dl), and IgE (60 IU/ml), and high antinuclear antibody titer (1/1,280) with a nuclear homogenous fluorescence pattern upon indirect immunofluorescence on HEp-2 cells (Table 1). The patient was seropositive for EBV (IgG) and seronegative for CMV; both viruses were negative by PCR, as was microscopy for Leishman bodies and acid-fast bacilli (Ziehl–Neelsen stain) in the bone marrow. Based on fever, splenomegaly, markedly increased ferritin (1,190 ng/ml), triglycerides (1,140 mg/dl), and soluble IL-2 receptor (sIL-2R; 3,202 U/ml) levels (Table 1), a formal diagnosis of HLH was established, although the patient lacked cytopenia, a characteristic of primary HLH (Henter et al., 2007). Methylprednisolone pulse therapy (30 mg/kg for 3 d consecutively), followed by oral dexamethasone (10 mg/m^2^/d and tapered over several weeks) and cyclosporine (3 mg/kg/d) were successful in controlling clinical symptoms and led to near-normalization of most laboratory parameters. She continued to do well on dexamethasone (1.25 mg/m^2^/d three times per week) and cyclosporine (target blood level of 100–200 ng/ml) up to the age of 9 mo, when she developed axillary BCG lymphadenitis (the aspirates were positive for acid-fast bacilli; Fig. 1 B). Accordingly, she was put on rifampicin, isoniazid, and ethambutol, which was switched after 2.5 mo to ethambutol, cotrimoxazole, and levofloxacin due to drug-induced hepatotoxicity. The infection is presently under control.

**Table 1.** Blood and immunological parameters of *NCKAP1L* patients

	Patient 1 (3 mo)	Patient 1 (8 mo)	Normal range	Patient 2 (9 yr)	Patient (11 yr)	Normal range
**Peripheral blood**						
White blood cells (×10^3^/µl)	85.5	7.4	6–17.5	3.46	10.6	5.4–9.7
Hemoglobin (g/dl)	9.5	11	9.5–13.5	6.60	12.4	11.3–13.4
RBC (x10^6^/µl)	3.16	4.84	3.5–5.5	3.02	5.65	4.00–5.20
MCV (fl)	95.9	82	80–100	66.4	62.5	77–90
MCH (pg)	30.1	27.5	27–31	22	18.9	25–30
Reticulocyte count (%)	2.4		0.5–1.5		0.3	0.5–1.5
Direct Coombs test	Negative	Negative		Negative	Negative	
Platelets (×10^3^/µl)	234	236	150–450	71	188	150–450
Neutrophils (×10^3^/µl)	21.4	2.1	1–8.5	1.66	2.84	2.5–5.95
Lymphocytes (×10^3^/µl)	51	3.3	4.0–10.5	1.38	5.07	1.23–2.76
Monocytes (×10^3^/µl)	7.7	1.8	0.2–1.2	0.42	0.87	0.10–1.11
Eosinophils (×10^3^/µl)	1.5		<0.81	0	0.12	0.04–0.19
**Lymphocyte subsets**^a^						
CD3 (×10^3^ cells/ml)	40.8	4.67	1.85–5.96	0.43	3.78	1–2.6
CD3/CD4 (×10^3^ cells/ml)	10.2	1.28	1.14–3.8	0.25	1.69	0.53–1.5
CD3/CD8 (×10^3^ cells/ml)	30.6	3.24	0.54–1.97	0.16	2.38	0.33–1.1
CD4/CD8 ratio	0.3	0.39	1.6–3.8	1.05	0.58	1.1–1.6
CD3/CD25 (×10^3^ cells/ml)	-	-		56	328	100–400
CD19 (×10^3^ cells/ml)	5.6	0.69	0.64–1.96	0.57	1.55	0.27–8.6
CD16/CD56 (×10^3^ cells/ml)	4.5	0.40	0.15–1.33	0.57	0.21	0.07–0.48
**% in CD4^+^ T cells**						
T reg (CD25^+^CD127^−^Foxp3^+^)	-	5.6	6.3–8.5*	-	7.4	7.6–9.9*
Naive (CD45RA^+^CCR7^+^)	-	33	74–90*	-	34	67–83*
CM (CD45RA^−^CCR7^+^)	-	20	8–18*	-	50	12–23*
EM (CD45RA^−^CCR7^−^)	-	34	2–5*	-	15	4–9*
TEMRA (CD45RA^+^CCR7^−^)	-	3	0–5*	-	1	1–2*
CD57^+^	-	6.5	1–2*	-	3.8	0–2*
CD69^+^	-	21	5–9*	-	5.0	5–7*
CD95^+^	-	69	12–24*	-	66.9	18–36*
HLA-DR^+^	-	7	2–4*	-	5.3	2–9*
PD1^+^	-	36	16–26*	-	34.3	17–22*
**% in CD8^+^ T cells**						
Naive (CD45RA^+^CCR7^+^)	-	6	60–85*	-	12	56–74*
CM (CD45RA^−^CCR7^+^)	-	1	0–6*	-	10	2–4*
EM (CD45RA^−^CCR7^−^)	-	5	2–10*	-	21	6–14*
TEMRA (CD45RA^+^CCR7^−^)	-	88	6–28*	-	57	13–30*
CD57^+^	-	49	0–16*	-	58	4–20*
CD69^+^	-	22	6–21*	-	17	12–24*
CD95^+^	-	61	0–18*	-	36	14–34*
HLA-DR^+^	-	30	5–17*	-	21	5–15*
PD1^+^	-	35	19–25*	-	25	16–31*
**% in CD19^+^ B cells**						
Naive (IgD^+^CD27^−^)	-	90	69–86*	-	93	64–86*
Switched memory (IgD^+^CD27^−^)	-	1	3–10*	-	3	5–17*
Unswitched memory (IgD^+^CD27^+^)	-	1	7–10*	-	3	5–15*
Transitional (CD24^+^CD38^+^)	-	10	8–19*	-	9	7–15*
CD21^−^	-	77	4–14*	-	2	2–5*
**% in monocytes**						
Classical (CD14^+^CD16^−^)	-	9	59–80*	-	72	80–85*
Transient (CD14^+^CD16^+^)	-	10	6–14*	-	17	3–5*
Resident (CD14^lo^CD16^+^)	-	75	5–12*	-	2	2–6*
**% in CD3^−^CD56^+^ NK cells**						
CD16^−^CD56^++^	-	18	2–8*	-	18	5–10*
NKp30^+^	-	26	69–86**	-	51	64–81*
NKp44^+^	-	8	0–11*	-	7	1–4*
NKp46^+^	-	96	96–98*	-	95	94–97*
NKG2C^+^	-	11	0–69**	-	10	7–19**
NKG2D^+^	-	98	97–98*	-	93	84–100*
CD57^+^	-	10	0–49*	-	28	10–23**
**Igs**						
IgA (mg/dl)	130	169	4–69	1,820	680	70–400
IgG (mg/dl)	2,581	1,680	196–560	15,900	22,500	700–1,600
IgM (mg/dl)	>1,000	9,550	25–93	1,340	1,160	40–230
IgE (IU/ml)	60	22.9	<15	358	197	0–500
**Serology**						
Antinuclear antibodies titer	-	Positive at 1/1,280 dilution	<1/80	Negative	-	<1/80
Anti-dsDNA (IU/ml)	-	Negative	<50	Negative	-	<200
Tetanus titer (IU/ml)	Protective	Protective	>0.15	Protective	Protective	>0.15
**Others**						
Ferritin (ng/ml)	1,190	241	36–100	14,280	76.9	21–274
Fibrinogen (mg/liter)	0.64	0.83	1.5–3.5	1.07	3.83	1.50–4.10
Triglycerides (mg/dl)	1,140	1,940	20–150	612	174	20–150
AST (U/liter)	141	84	6–50	301	102	5–34
ALT (U/liter)	21	35	20–60	313	121	5–55
Total bilirubin (µmol/liter)	6.84	3.42	3.4–17.1	15.1	5.1	3.4–17.1
Conjugated	3.42	-	<5.9	14.2	-	<5.9
Unconjugated	3.42	-	<17.1	0.9	-	<17.1
Alk Phos (IU/liter)	709	680	110–320	174	221	141–460
Haptoglobin (g/liter)	-	-	-	0.47	-	<2.7
LDH (IU/liter)	-	2,835	500–920	948		432–700
ESR (mm/h)	1	20	0–10	28	25	0–15
CRP (mg/liter)	1	1	<6	208	5	< 8
sIL-2R (U/ml)	-	3,202	223–710	-	1,701	223–710
IFN-γ (pg/ml)	-	5.3	1.7–66.5^b^	-	339	0.3–11.0^c^
IL-10 (pg/ml)	-	48.3	0–2.5^b^	-	12.6	0–10.3^c^
IL-18 (pg/ml)	-	3,698	17–145^b^	-	2,130	266–1,300^c^
IP-10 (pg/ml)	-	228	25–68^b^	-	518	37–245^c^
IL12p40 (pg/ml)	-	676	0.0–74^b^	-	152	0.0–65^c^

“-” indicates test not done. Normal-range values are either those of the local diagnostic laboratory and/or reported from Gregory and Andropoulos (2012). * and ** correspond to 5th and 95th percentile of values obtained from eight age-matched controls and, in some rare cases, from three age-matched controls, respectively. Alk Phos., alkaline phosphatase; ALT, alanine transaminase; AST, aspartate transaminase; CRP, C-reactive protein; dsDNA, double-stranded DNA; ESR, erythrocyte sedimentation rate; LDH, lactate dehydrogenase; MCH, mean corpuscular hemoglobin; MCV, mean corpuscular volume; T reg cells, regulatory T cells.

a Normal range for absolute numbers of lymphocyte subsets were from age-matched routine clinical lab values.

b Normal range shows 5th and 95th percentile of serum cytokine levels calculated among 22 healthy subjects.

c Normal range shows 5th and 95th percentile of plasma cytokine levels calculated among 10 healthy subjects.

Patient 2 is an 11-yr-old son of healthy first-cousin parents from Saudi Arabia (Fig. 1 A), whose siblings were also all equally healthy and who, like the previous patient, received a standard set of vaccinations at birth/early childhood (including BCG at birth). At the age of 5 yr, the patient started to have recurrent otitis media, fever, and sinopulmonary infections that responded only partially to antimicrobial therapies. A chest computed tomographic scan showed right-side basal bronchiectasis and focal area of oligemia (Fig. 1 C). He underwent adenotonsillectomy and bilateral insertion of tympanostomy tubes at the age of 7 yr. At the age of 9 yr, he presented with recurrent viral illnesses, including upper respiratory infections, gastroenteritis, and infectious mononucleosis. Clinical examination showed hepatosplenomegaly (Fig. 1 D). The laboratory workup showed pancytopenia (white blood cells 3,460 cells/µl, hemoglobin 6.60 g/dl, platelets 71,000 cells/µl, neutrophils 1,660 cells/µl, lymphocytes 1,380 cells/µl), mild to moderate anisopoikilocytosis, and moderate transaminitis (aspartate transaminase and alanine transaminase at 301 and 313 U/liter, respectively; Table 1). His bone marrow biopsy showed histiocytes with phagocytosis of erythrocytes, lymphocytes, and platelets (Fig. 1 E). The patient displayed low-titer EBV viremia (<500 copies/ml with no treatment) and was seropositive (IgG) for the virus, while CMV serology and PCR were both negative. Other biological findings included massive IgG, IgA, and IgM hyperimmunoglobulinemia (15,900, 1,820, and 1,340 mg/dl, respectively), normal IgE, and negative autoantibodies. Hematoxylin and eosin and CD68 staining of liver tissue revealed sinusoidal dilatation with hemophagocytic histiocytosis (Fig. 1, F and G). The presence of hypertriglyceridemia (612 mg/dl), hyperferritinemia (14,280 ng/ml), and a mildly increased sIL-2R at 1,701 U/ml (Table 1) completed the picture for a formal diagnosis of HLH (Henter et al., 2007), for which he was started on the HLH-2004 protocol (Bergsten et al., 2017). Despite completing the treatment course, he continued to have high liver enzymes with no clear etiology. He was given another course of etoposide and dexamethasone with no significant improvement. Currently, he is off therapy with mild transaminitis.

### Identification of a candidate gene

Given the unusual symptomatology of the patients somewhat deviating from typical familial HLH, we aimed directly at whole-exome sequencing for the identification of the culprit gene. All family members (patients, both their parents, and several siblings in family 2) were whole-exome sequenced. Both families displayed a variant in *NCKAP1L* segregating with the disease with an autosomal recessive inheritance model—i.e., homozygous in the patient while WT or heterozygous in all other family members. Both variants were confirmed by targeted Sanger sequencing (Fig. 1 H). No other deleterious variants were found in genes associated with primary HLH or primary immunodeficiencies (Picard et al., 2018). Neither identified variant was present in the Exome Variant Server, Exome Aggregation Consortium, Genome Aggregation Database, our in-house database, and various other databases. (Table S1 reports other variants found in patient 1; no additional variants were found in patient 2.) In patient 1, a c.421G>T (p.V141F; NM_005337; chr12:54902230G>T) missense variant was identified (Fig. 1 H), while the variant c.2862+1G>A (NM_005337; chr12:54926035G>A) in patient 2 abolished the donor splice site in intron 26 (Fig. 1 H), leading to the skipping of the 26th exon, as confirmed by RT-PCR (Fig. 1 I) and RNA sequencing (RNA-seq; data not shown) experiments. In silico analyses predicted the variants to be damaging with scores of 0.008 (scale-invariant feature transform), 0 (likelihood ratio test), 4.09 (Genomic Evolutionary Rate Profiling [GERP]++), 0.954 (polymorphism phenotyping v2), and 26.2 (Combined Annotation-Dependent Depletion) for p.V141F, and 4.17 (GERP++), 4.564 (PhyloP), and 25.8 (Combined Annotation-Dependent Depletion) for c.2862+1G>A (Adzhubei et al., 2010; McKenna et al., 2010; Yang and Wang, 2015). The Mutation Taster tool predicted both variants to be disease causing and, according to the American College of Medical Genetics and Genomics guidelines, c.421G>T and c.2862+1G>A can be classified as PM2 (moderate pathogenic) and PVS1 (pathogenic very strong), respectively (Richards et al., 2015; Schwarz et al., 2014). Moreover, the NCKAP1L protein was undetectable by Western blot (and proteomics analysis; see below) in both patients, as was WAVE2, which requires interaction with NCKAP1L for stabilization (Fig. 1 J). Therefore, both patients harbor recessive loss-of-function mutations in *NCKAP1L*.

### Effect of *NCKAP1L* mutations on protein structure

Structural modeling confirmed the deleterious effects of both mutations on protein structure. The model structure of NCKAP1L was built in the context of the WAVE complex (Chen et al., 2014) using NCKAP1 as a template (Fig. 1 K). NCKAP1L binds to CYFIP1 to form the support for the large complex, while the Abl interactor 2 protein (ABI2, shown in salmon color) has proximal contacts with both the helices around the V141F mutation (shown in red) and the exon 26 helix region (shown in purple). The V141F mutation is located in the N terminus of NCKAP1L. Given that phenylalanine has a larger side-chain group than valine, the mutation would result in severe atomic clashes (Fig. 1 L vs. Fig. 1 M) leading to the reorganization of the domain, thus preventing the interaction with other components of the WAVE complex. With regard to the c.2862+1G>A mutation in patient 2, once exon 26 is missing, the resulting protein is unable to make several interactions with ABI2, thereby producing a dysfunctional protein complex (Fig. 1 N vs. Fig. 1 O). Hence, structural modeling does support the observed absence of detectable NCKAP1L and WAVE2 proteins in patient cells (Fig. 1 J).

### Immunological phenotype of NCKAP1L-deficient patients

**Figure 2.**
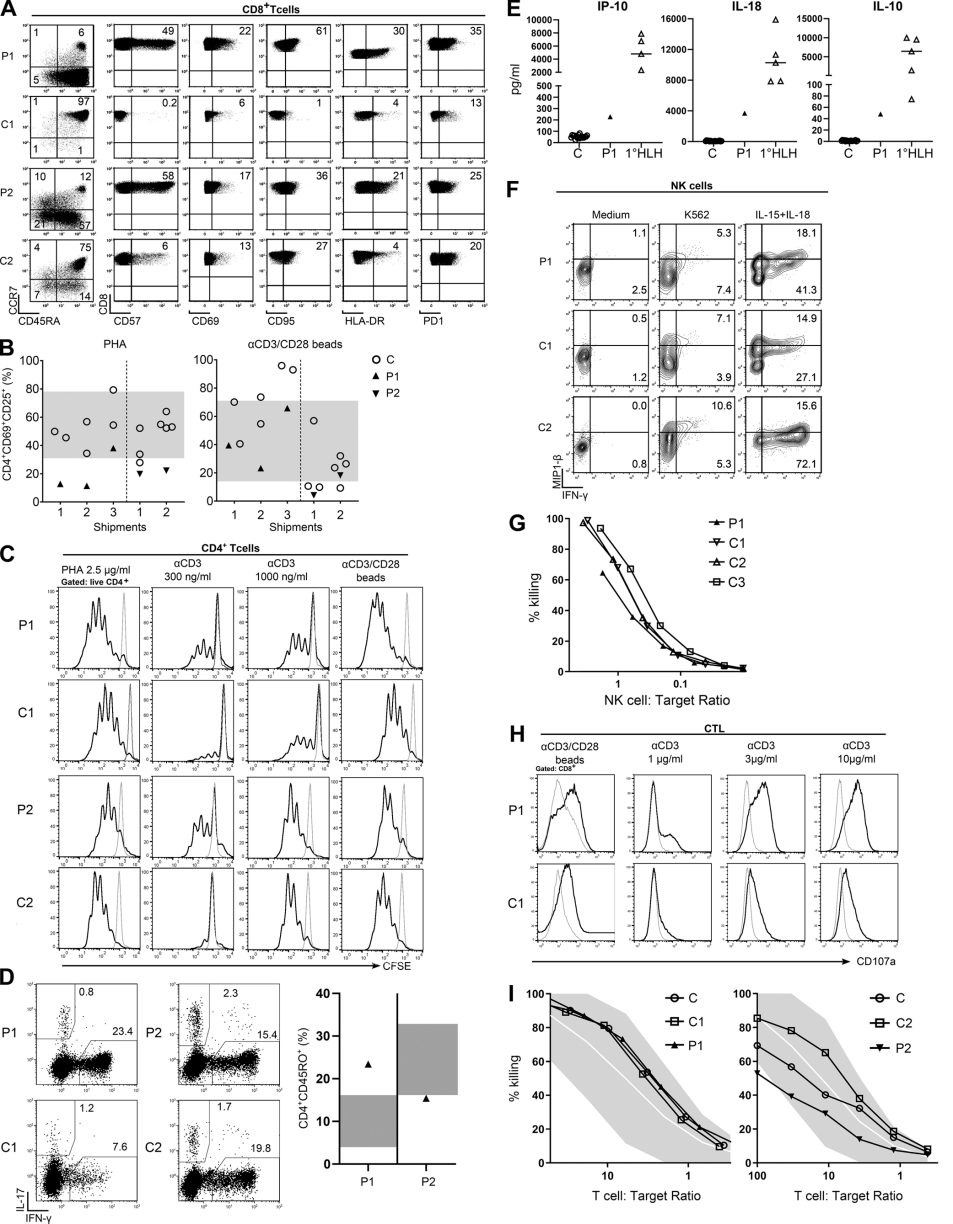
**Immunophenotyping, T and NK cell stimulation, proliferation, degranulation, and cytotoxicity.** **(A)** Immunophenotyping of CD8^+^ T cells: gating of naive (CD45RA^+^, CCR7^+^), CM (CD45RA^−^, CCR7^+^), EM (CD45RA^−^, CCR7^−^) and EM RA (CD45RA^+^, CCR7^−^) populations and expression of CD57, CD69, CD95, HLA-DR, and PD-1 in CD8^+^ T cells. In each dot plot, the numbers indicate the percentage of the gated populations. The experiments were repeated on three separate samples from each patient (unless otherwise specified). The shown control sample is one representative example of eight age-matched controls analyzed for each patient. **(B)** T cell activation markers: PBMCs were stimulated with PHA or anti-CD3/anti-CD28–coated beads for 20 h, and up-regulation of CD69 and CD25 was determined by flow cytometry. Summarized results of determinations done on cells from three different shipments of patient 1 (P1, filled, up facing triangles) or two shipments of patient 2 (P2, filled, down-facing triangles) are compared with reference values obtained from 50 in-house controls (gray area indicates 5th–95th percentile) and travel controls (C, open circles). **(C)** T cell proliferation. Histograms show CFSE dilution of live CD4^+^ T cells after 5 d of stimulation of PBMC from patient 1 (P1) or her father (C1; upper panels) or patient 2 (P2) or his father (C2; lower panels) with PHA, plate-bound anti-CD3, or plate-bound anti-CD3/anti-CD28. Gray line, medium; black line, stimulated. **(D)** Ex vivo T cell cytokine expression. Dot plots showing IL-17 and IFN-γ expression of patient 1 (P1) and patient 2 (P2) CD4^+^CD45RO^+^ memory T cells together with a representative age-matched control (C1 and C2, respectively; left panels). Summary graph shows the percentage of IFN-γ^+^ memory T cells from patient 1 (filled, up-facing triangle) and patient 2 (filled down-facing triangles) compared with reference values obtained from in-house healthy controls (*n* = 21, 0–2 yr old; *n* = 90, >10 yr old; gray area indicates 5th–95th percentile). **(E)** Serum cytokine levels of IFN-γ–regulated cytokines/chemokines. Summarized results show levels of the indicated cytokine or chemokine of patient 1 (P1, filled up-facing triangles) in comparison with healthy controls (C, open circles) and patients with primary HLH (open up-facing triangles). **(F)** NK cell cytokine production. PBMC from patient 1 (P1), an age-matched shipping control (C1), and an internal in-house control (C2) were cultured with IL-15 and IL-18 for 24 h or in the presence of K562 for 6 h before intracellular IFN-γ and MIP-1β staining. Dot plots show cytokine expression of CD56^+^CD3^−^ NK cells. **(G)** NK cytotoxicity. Lytic activity of overnight rested PBMCs on ^51^Cr-labeled K562 cells. The percentage of NK cells among PBMCs as determined by flow cytometry was used to calculate the NK:target cell ratio. Representative graph of two experiments performed with two different blood shipments shows patient 1 (P1) compared with her father (C1), mother (C2), and an unrelated control (C3). **(H)** CTL degranulation. Histograms show CD107a expression of T cell blasts from patient 1 (P1) or her father (C1) kept for 24 h in medium without IL-2 followed by stimulation with anti-CD3/CD28 beads or the indicated concentrations of plate-bound anti-CD3. Gray line: medium, black line: stimulated. **(I)** CTL cytotoxicity. Lytic activity of long-term T cell blasts overnight rested from IL-2 on ^51^Cr-labeled K562 cells. The percentage of CD8^+^ cells determined by flow cytometry was used to calculate the T cell:target ratio. Representative graph of two experiments shows patient 1 (P1) in comparison with her father (C1) and an unrelated control (C) or patient 2 (P2) in comparison with his father (C2) and an unrelated control (C).

**Figure S1.**
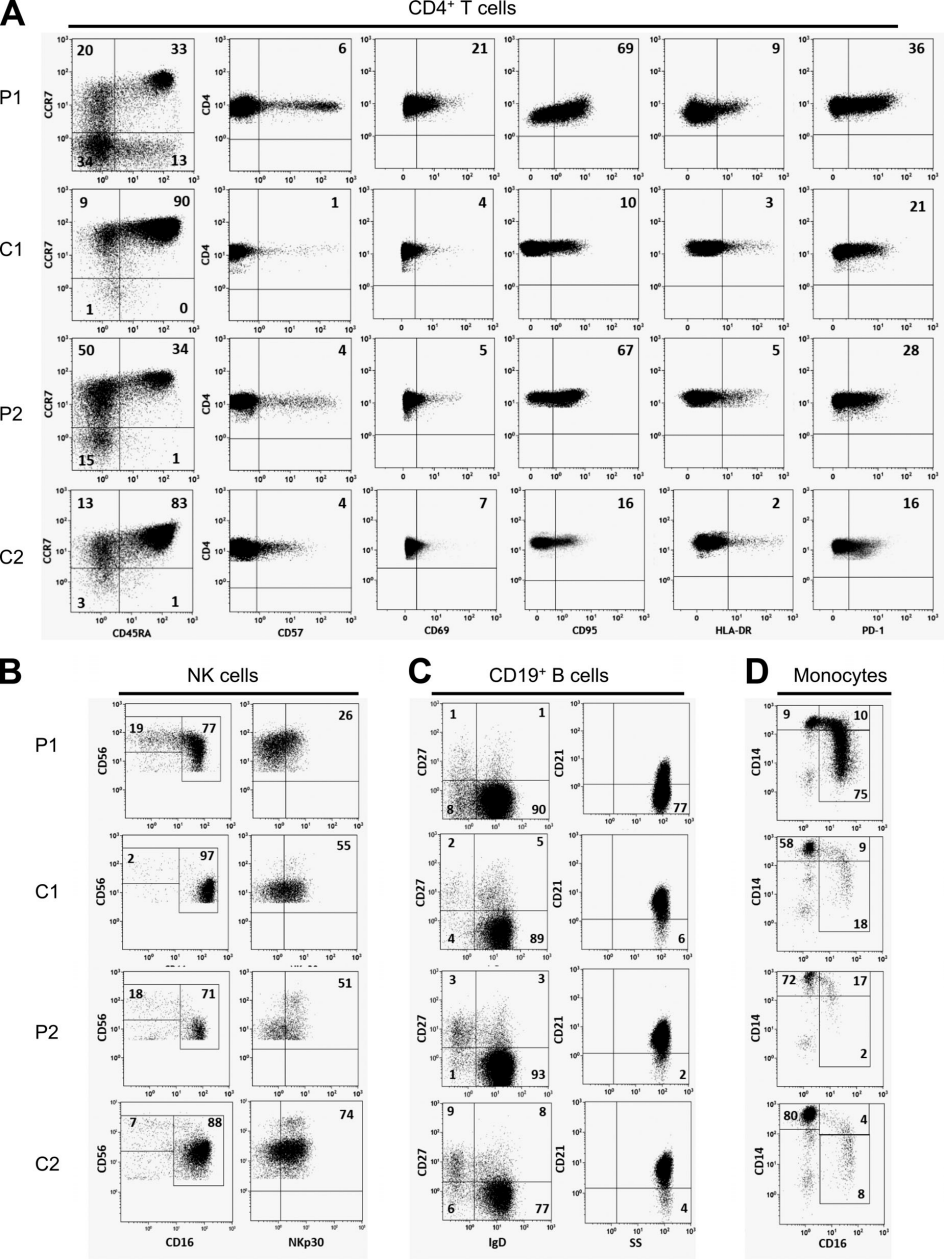
**Immunophenotyping of CD4^+^ T cells, NK cells, B cells, and monocytes by flow cytometry.** **(A)** CD4^+^ T cells: gating of naive (CD45RA^+^, CCR7^+^), CM (CD45RA^−^, CCR7^+^), EM (CD45RA^−^, CCR7^−^) and EM RA (CD45RA^+^, CCR7^−^) populations and expression of CD57, CD69, CD95, HLA-DR, and PD-1 in CD4^+^ T cells. **(B)** CD19^+^ B cell phenotype; naive (IgD^+^CD27^−^), switched (IgD^−^CD27^+^), and unswitched (IgD^+^CD27^+^) memory subsets (left) and CD21 expression in CD19^+^ B cells (right). **(C)** NK cells (CD3^−^CD56^+^cells) phenotype; CD56^bright^CD16^−/lo^ and CD56^+^CD16^+^ NK cells (left) and NKp30 expression in total NK cells (right). **(D)** Monocyte subsets identified as classical monocytes (CD14^+^CD16^−^), transient (CD14^+^CD16^+^), and resident (CD14^lo/−^CD16^+^). In each dot plot, the numbers indicate the percentage of the gated populations. Only one representative control is shown in the figure, with the exception of NK cell subsets, which were compared with three age-matched controls; the other lymphocyte subsets were measured against at least eight age-matched controls (for each patient).

Compared with age-matched controls, we observed an increased number of CD8^+^ T cells with an inversion of the CD4/CD8 ratio and an activation phenotype characterized by increased percentages of all memory subsets in CD8^+^ T cells: central memory (CM; CD45RA^−^CCR7^+^), effector memory (EM; CD45RA^−^CCR7^−^), and terminally differentiated EM cells (TEMRA; CD45RA^+^CCR7^−^; Fig. 2 A and Table 1). This increase was associated with increased expression of activation and senescence markers (CD57, CD69, CD95, HLA-DR, and PD1; Ammann et al., 2017; Fig. 2 A). Similar results were observed in CD4^+^ T cells, although to a lesser extent (Fig. S1 A). Compared with patient 1, the T cell phenotype was less pronounced in patient 2 (Fig. 2 A and Fig. S1 A). In both patients’ B cell compartments, we observed a slight increased percentage of naive cells associated with a decreased percentage of memory subsets (switched and unswitched; Table 1). However, and only in patient 1, we observed an abnormal proportion of CD19^+^CD21^−^ B cells, a population that has been associated with autoimmune diseases such as systemic lupus erythematosus (Wehr et al., 2004), in line with high titers of antinuclear antibodies found in this patient (Table 1 and Fig. S1 B).

Among NK cells, CD56^hi^CD16^−/lo^ NK cells were increased, and natural cytotoxicity receptor expression was altered with a lower expression of NKp30 in both patients, which could correspond to abnormal differentiation and function of these NK cells (Fig. S1 C). Moreover, in patient 1, CD16^+^NK cells were characterized by a continuum in CD56 expression without possible distinction between CD56^+dim^ and CD56^+bright^ cells, and in patient 2, a higher expression of CD57 was observed (Fig. S1 C and Table 1). Finally, the percentage of classical (CD14^+^CD16^−^) monocytes was reduced in favor of inflammatory monocytes (CD14^+^CD16^+^ transient and/or CD14^lo^CD16^+^ resident monocytes) in both patients (Fig. S1 D and Table 1).

### Human NCKAP1L deficiency is associated with impaired early T cell activation but normal NK and cytotoxic T lymphocyte (CTL) cytotoxicity

CD4^+^ T cells from NCKAP1L-deficient patients consistently showed reduced up-regulation of CD69 and CD25 after stimulation with PHA, while they reacted normally to anti-CD3/CD28-coated beads (Fig. 2 B). This was also observed independently of the differences in memory and naive T cell frequencies of patients and controls and the stimulation strength (data not shown). Nevertheless, T cells from both patients generally proliferated normally after stimulation with PHA, or different doses of plate-bound or low doses of plate-bound anti-CD3 in combination with soluble anti-CD28 (Fig. 2 C). Thus, while early T cell activation events were impaired, this did not impair the overall in vitro proliferation response. Moreover, the cytokine profile of memory T cells measured in both patients showed an elevated frequency of IFN-γ–producing cells in patient 1 (Fig. 2 D), consistent with his elevated serum IL-18 and IP-10 levels (Table 1 and Fig. 2 E). Although patient 2 also showed elevated plasma IFN-γ–dependent cytokines/chemokines (Table 1), ex vivo IFN-γ production by memory T cells was within the normal range (Fig. 2 D). This discrepancy could stem from different ongoing inflammatory processes at the time of sampling. Of note, elevated serum levels of IP-10, IL-18, and IL-10 in patient 1 in contrast to controls were still not as pronounced as in patients with an active primary HLH (Fig. 2 E).

Considering the HLH-like clinical phenotype of the patients, we carefully evaluated degranulation and cytotoxic responses of NK cells and activated CD8^+^ T cells (CTL). NK cells from patient 1 could be normally activated, expressing levels of MIP-1β and IFN-γ upon stimulation with K562 target cells and a combination of IL-15 and IL-18 comparable to controls (Fig. 2 F). The degranulation of patient 1 NK cells had a higher basal level that did not further increase when stimulated with K562 target cells. NK cells from patient 2 did not show abnormalities compared with the travel control; therefore, a general NK degranulation problem was not observed (data not shown). Moreover, the ability of patient 1 NK cells to lyse K562 target cells as measured in a 4-h ^51^Cr release assay was comparable to controls (Fig. 2 G).

The degranulation capacity measured in CTL of patient 1 and patient 2 upon strong stimulation with anti-CD3/28 beads resulted in normal or even increased levels of surface CD107a expression. Additionally, patient 1 CTL degranulation was not decreased compared with controls when tested under limiting conditions with titrated concentrations of plate-bound anti-CD3 (Fig. 2 H). Likewise, T cell lines from both patients showed a normal killing capacity (Fig. 2 I).

Overall, NCKAP1L deficiency impaired early T cell activation but did not affect T and NK cell cytotoxicity under the given experimental conditions.

### Impaired actin polymerization in *NCKAP1L*-deficient T cells is associated with impaired synapse and leading edge formation

**Figure 3.**
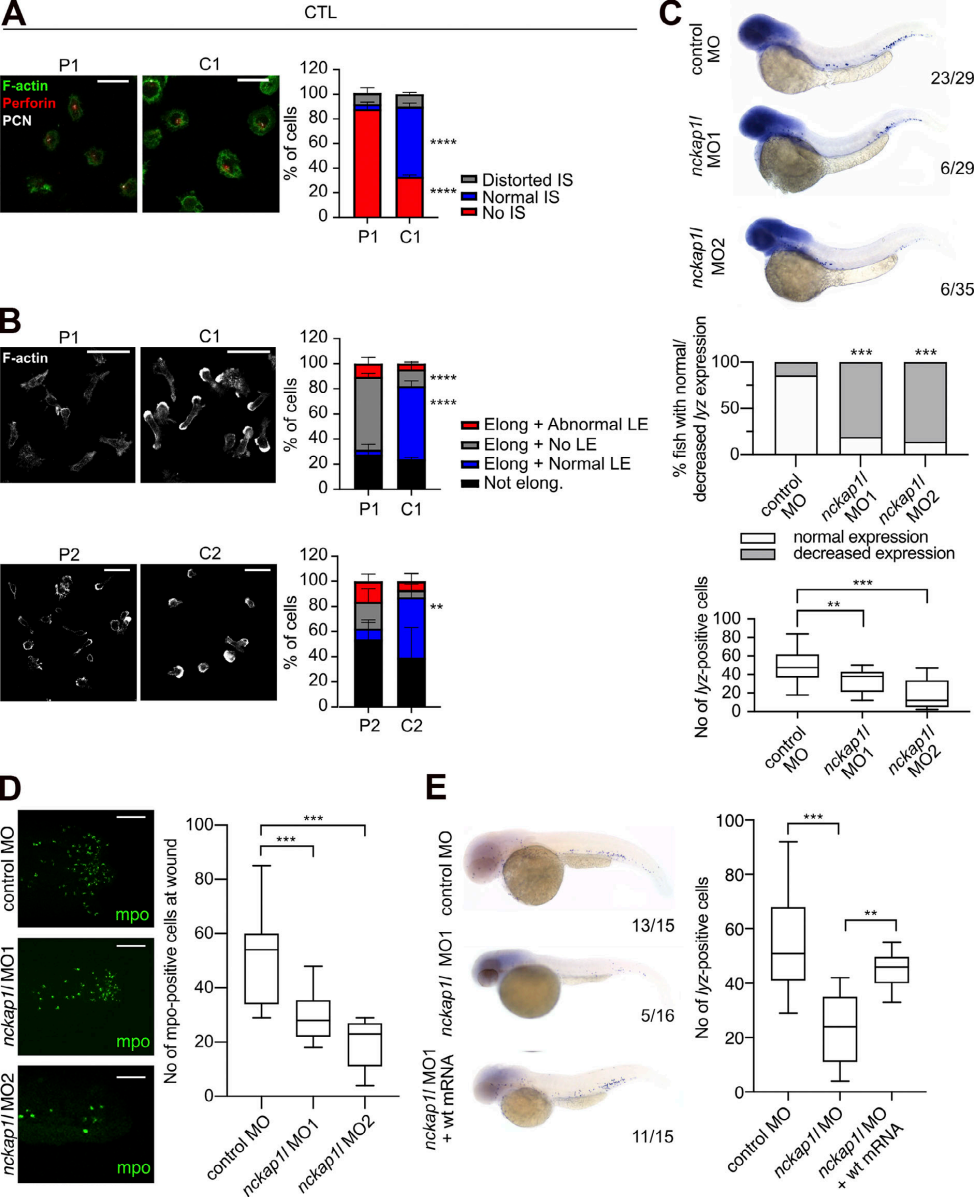
**Functional consequences of NCKAP1L deficiency in T cells and neutrophils.** **(A)** IS formation. Fluorescence microscopy of long-term T cell blasts from patient 1 (P1) and her father (C1), placed on top of a CD3-coated surface for 6 min. Green, F-actin; white, centrosome (PCN); red, perforin. Representative images of two experiments (left panels). Bar graphs showing percentage of cells with a distorted, abnormal, or no IS formation in a total of 30–40 cells per individual per experiment (right panel). Two-way ANOVA with Sidak’s multiple comparison test; ***, P < 0.001; ****, P < 0.0001; scale bar, 15 µm. **(B)** Leading edge formation and migration. Fluorescence microscopy of long-term T cell blasts of patient 1 (P1) and her father (C1) or patient 2 (P2) and his father (C2) while crawling on an ICAM-1–coated surface (left panels). White, F-actin. Representative images of two or three experiments. Bar graphs showing percentage of elongated (Elong) cells with normal, no, or abnormal leading edge (LE) or not elongated of a total of 50–80 cells per individual per experiment. Two-way ANOVA with Sidak’s multiple comparison test, **, P < 0.01; ****, P < 0.0001; scale bar, 30 µm. **(C)** WISH of *lyz* at 48 hpf in control and MO-injected zebrafish embryos. Shown are (from top to bottom) representative images for each phenotype (top), graphs displaying the percentage of fish with normal vs. decreased expression (middle) assessed according to the numbers of neutrophils in each phenotype (bottom). Numbers indicate the amount of fish with normal expression/total amount of fish from three biological replicates. **(D)** Fewer neutrophils are found at the wound site after tail fin wounding. Representative pictures and corresponding quantitation of neutrophils as measured by mpo antibody staining indicate reduced numbers of neutrophils at the wound in *nckap1l* morphants (*n* = 17 for MO1, *n* = 11 for MO2) compared with control injected embryos. Scale bar, 100 µm. **(E)** Co-injection of wt mRNA rescues neutropenia in *nckap1l* morphants. Shown are representative images (left) and graphs displaying the number of *lyz+* cells after WISH from control, *nckap1l* MO, and *nckap1l* MO + wt mRNA coinjected transgenic embryos (right). Lateral views are shown, with anterior to the left, dorsal up. Numbers indicate the amount of fish with normal expression/total amount of fish assessed by the quantified numbers of neutrophils. Summarized data from *n* = 3 biological replicates with at least *n* = 4 embryos per experiment are shown. An ordinary one-way ANOVA was performed for multiple comparisons. Error bars are shown as ± standard deviation; **, P < 0.01; ****, P < 0.0001.

Since the WAVE complex is one of the nucleation promoting factors activating the Arp2/3 complex and allowing the polymerization of F-actin, we sought to study the effect of the loss of NCKAP1L on this tightly regulated machinery. The basal F-actin content in resting CD4^+^ and CD8^+^ T cells from patient 1 did not differ from controls (data not shown). However, abnormal actin polymerization became evident when long-term CD8 T cell lines from the patient were allowed to spread on an anti-CD3–coated surface before fixation and F-actin staining. While control cells were able to assemble a dense ring of F-actin around the docking centrosome (marked by pericentrin), patient T cells exhibited a dysmorphic structure, lacking the characteristic synapse morphology (Fig. 3 A). Nevertheless, we detected perforin clustering around the centrosome in both patient and control T cells (Fig. 3 A), consistent with their intact degranulation capacity (Fig. 2 I).

Actin remodeling is also required for T cell movement. When T cells are placed on top of an ICAM-1–coated surface, their displacement is associated with actin-rich protrusions at the leading edge. While this could readily be observed in control CD8 T cell blasts, these protrusions were not detected in patient 1 and only poorly detected in patient 2 T cells (Fig. 3 B), despite their ability to adhere and elongate.

These results indicate that the deficiency of *NCKAP1L* in patient T cells has an impact on the dynamics by which the actin cytoskeleton rearranges, either to form a proper immune synapse (IS) or to adopt the morphological structures that allow them to migrate. This cellular phenotype was more pronounced than expected by the functional degranulation and cytotoxicity assays. This may be explained by the fact that the need for a proper IS that enables docking to the target cell and efficient degranulation and killing might not be reflected in vitro, where shear forces are minimal since the cells are cultured together with their target cells in a confined space.

Of note, other known actinopathies that affect proper immune/lytic synapse formation impact CTL and NK degranulation and cytotoxicity to different extents. ARPC1B and Wiskott–Aldrich syndrome protein (WASP)–interacting protein (WIP) deficiencies lead to decreased degranulation due to abnormal IS formation. NK cells from DOCK8- and DOCK2-deficient patients still form normal conjugates with their targets, but the compromised RAC activation leads to a defect in the lytic synapse and thus a reduced killing capacity (Kearney et al., 2017; Sakai et al., 2013). In Coronin 1A (CORO1A) deficiency, NK cytotoxicity ranged from normal to reduced (Mace and Orange, 2014; Yee et al., 2016), while CTL degranulation or killing was normal (Yee et al., 2016). In WAS patients, degranulation is mostly normal and CTL cytotoxicity ranges from mildly defective to preserved (De Meester et al., 2010). Overall, cytotoxicity defects in actinopathies cover a wide spectrum, with many of them being subtle, and there is no clear evidence that such mild defects can cause the pathophysiological sequence of events observed in primary HLH or HLH-like syndromes.

All in all, the observed cellular defects are largely consistent with the immune function of NCKAP1L identified in murine studies (see below). They likely explain the combined susceptibility to infection and immune dysregulation phenotype in the patients, which are also characteristic for other immunodeficiencies affecting actin cytoskeleton remodeling (Dobbs et al., 2015; Janssen et al., 2016; Moulding et al., 2013; Schober et al., 2017).

### Combined transcriptomics and proteomics analyses reveal an HLH-like immune activation signature

**Figure 4.**
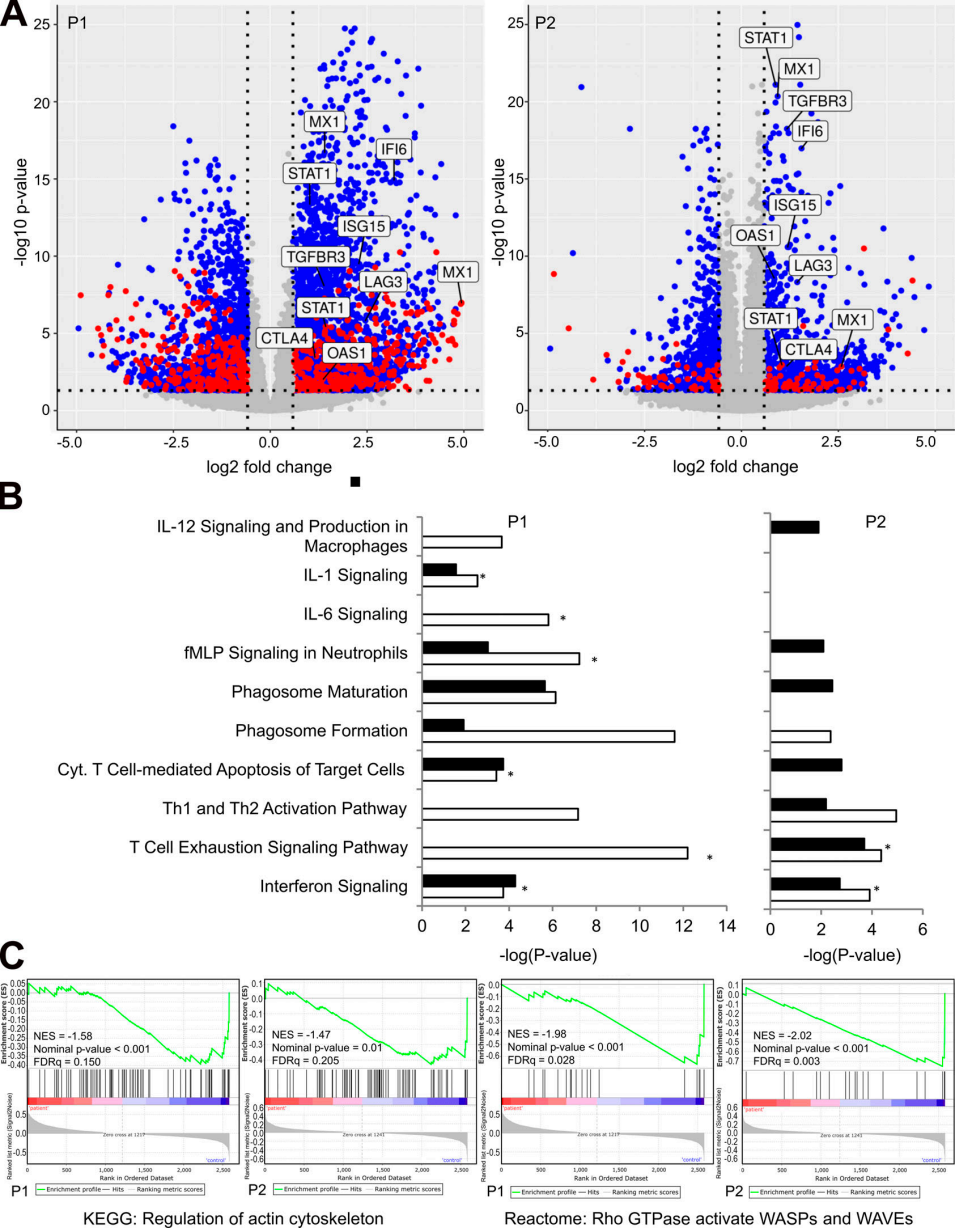
**Transcriptomic and proteomic analyses.** **(A)** Volcano plots showing up- and down-regulated genes and proteins in patients 1 and 2 using fold change and P value cutoffs of 1.5 and 0.05, respectively. The labels correspond to genes/proteins of the IFN and T cell exhaustion pathways. Only genes/proteins common to both patients are highlighted. **(B)** Canonical pathways deregulated in patients at the transcript (white bars) and proteome (black bars) levels. Positive z-scores are highlighted with an asterisk and correspond to predicted activation of the signaling pathway. **(C)** Gene set enrichment analysis plots showing negative enrichment of actin cytoskeleton regulation and WASPs/WAVEs activation by Rho GTPase pathways. fMLP, N-formyl-methionyl-leucyl-phenylalanine; NES, normalized enrichment score; Cyt., cytotoxic; Th, T helper; FDRq, false discovery rate q-value; KEGG, Kyoto Encyclopedia of Genes and Genomes.

**Figure S2.**
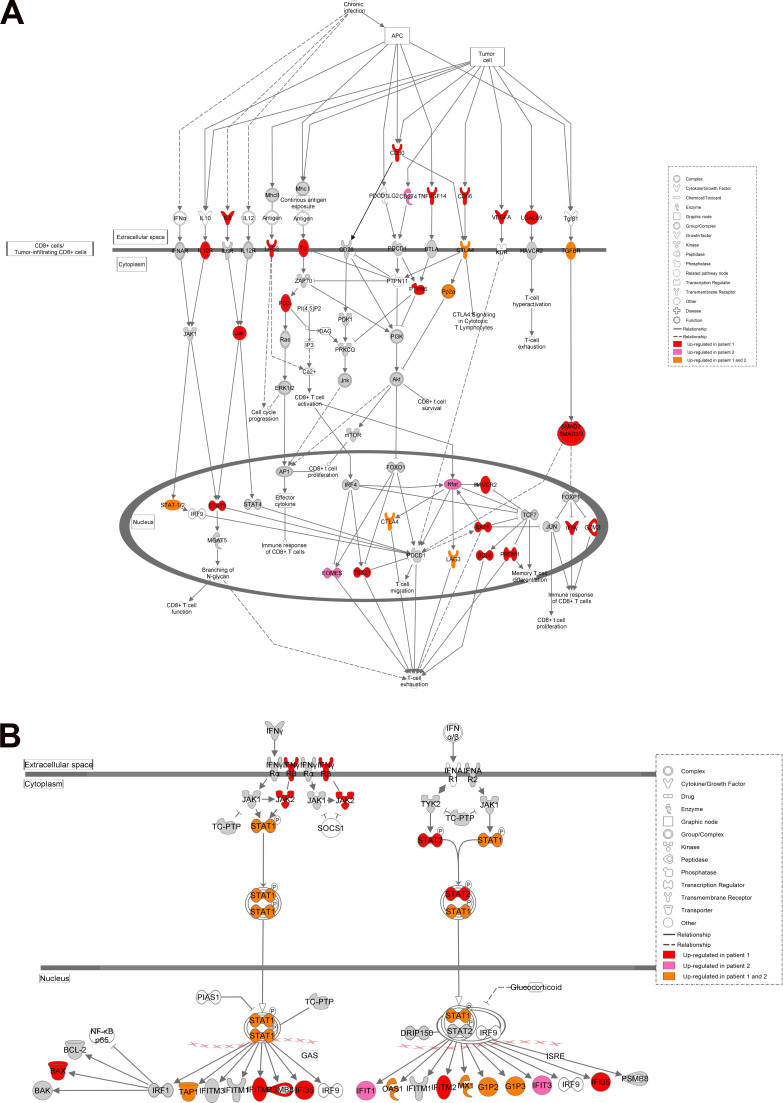
**Up-regulated canonical signaling pathways in patients 1 and 2.** **(A)** T cell exhaustion pathway. **(B)** IFN pathway. Both up-regulated genes and proteins were considered in this analysis.

Gene expression analyses by transcriptome and proteome characterization in peripheral blood mononuclear cells (PBMCs) of patients and healthy controls revealed deregulation of several canonical immune-related cellular pathways. Among these, IFN and T cell exhaustion signaling pathways were shown to be activated in both patients (Fig. 4, A and B; and Fig. S2). Indeed, IFN-γ–regulated cytokines such as IL-18 and IP-10 were found increased in both patients (Table 1). Patient 1 showed deregulation of additional immune pathways involving T cells, macrophages, neutrophils, and pro-inflammatory cytokines, as well as phagocytosis, all hallmarks of active HLH. In accordance with the defect in actin polymerization (see above), gene set enrichment analysis (GSEA) revealed that the regulation of the actin cytoskeleton gene signature was down-regulated in both patients (Fig. 4 C, left panels). Moreover, in line with the NCKAP1L deficiency, the signaling pathway of WAVE complex activation by Rho guanosine triphosphatase (GTPase) was confirmed to be down-regulated as well (Fig. 4 C, right panels). Together, these data suggest a strong immune activation associating inflammatory and T cell responses to decreased actin cytoskeleton regulation, which is presumably linked to a reduced activation of the WAVE complex pathway. It is interesting to note that in certain genetic forms of HLH, T cell exhaustion has been linked to a less severe form of the disease (Kögl et al., 2013). This might explain why, although serious, the disease severity in our two patients has not (yet) required stem cell transplantation. Finally, by equivalent proteomic and transcriptomic profiling of three cases of familial hemophagocytic lymphohistiocytosis type-5, we indeed identified the same deregulated pathways, except IL-1 signaling and N-formyl-methionyl-leucyl-phenylalanine signaling in neutrophils (data not shown), further highlighting a similar pathophysiology of our disease as compared with bona fide genetic HLH.

### In vivo functional impact of mutant *NCKAP1L*

**Figure S3.**
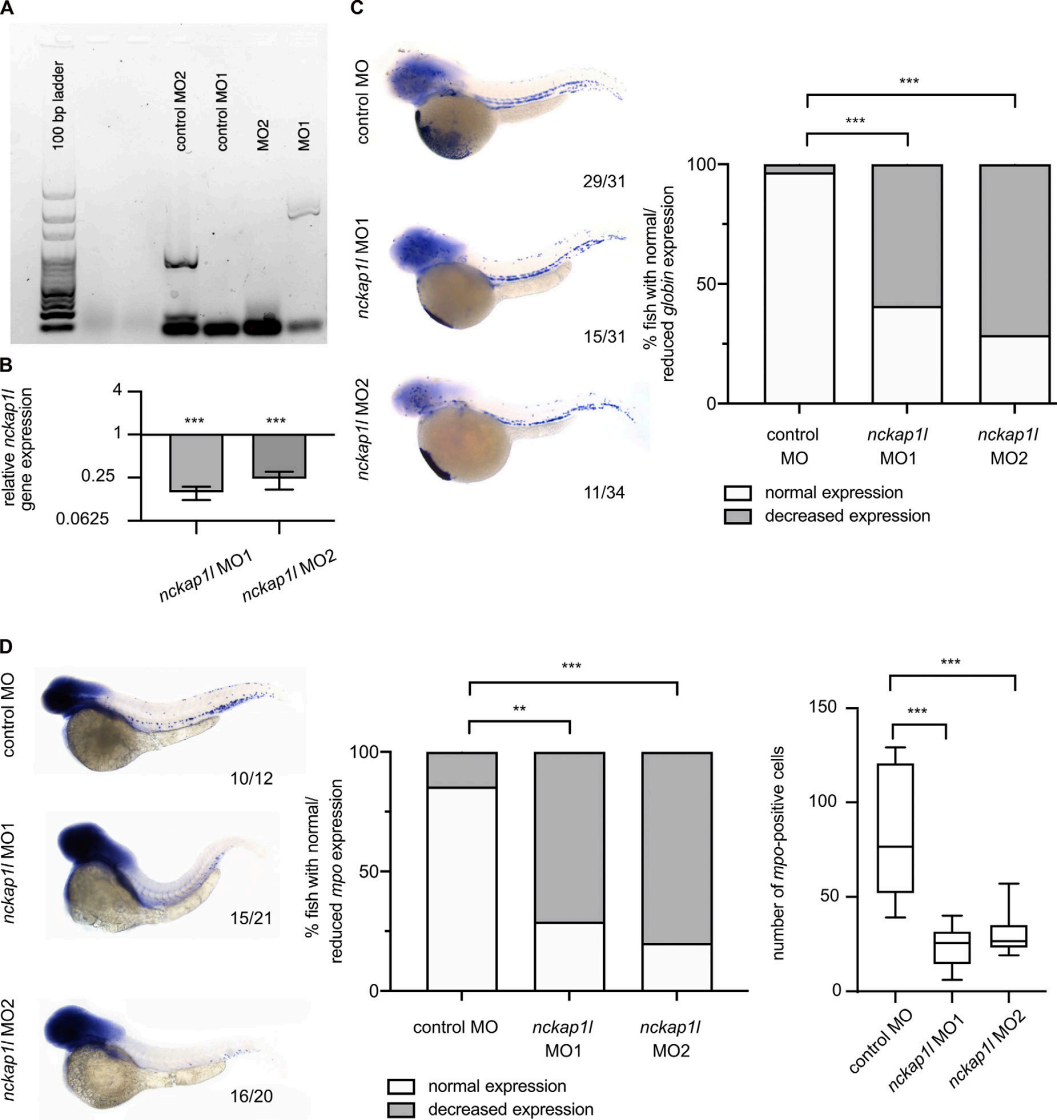
**Knockdown of zebrafish *nckap1l* leads to anemia and neutrophil reduction.** **(A)** Splice modification upon MO knockdown as shown by gel electrophoresis after RT-PCR using primers around the respective splice sites in control injected embryos (control MO1 and control MO2) and MO-injected embryos (MO1 and MO2). **(B)** Quantitative RT-PCR indicates knockdown of *nckap1l* upon MO injection. *Gapdh* was used as an internal control. Y axis is represented as antilog. **(C and D)** WISH of *globin* (C) and *mpo* (D) at 48 hpf in control and MO-injected zebrafish embryos. Shown are representative images for each phenotype (left, C and D), graphs displaying the percentage of fish with normal vs. decreased expression (middle), which in D (right) are assessed according to the numbers of neutrophils. Numbers indicate the amount of fish with normal expression/total amount of fish from three (C) or two (D) biological replicates. An ordinary one-way ANOVA was performed for multiple comparisons. Error bars are shown as ± SD; **, P < 0.01; ***, P < 0.001.

The zebrafish is an established model for studies on hematopoiesis, on developmental biology, and in generating experimental models of various human diseases (Jagannathan-Bogdan and Zon, 2013; Konantz et al., 2019). To investigate the role of *nckap1l* in zebrafish embryonic development, we performed in vivo loss-of-function experiments by treating zebrafish embryos with two different antisense morpholino oligonucleotides (MOs) to inhibit *nckap1l* premRNA splicing. We found that transcripts in morphants were indeed misspliced (Fig. S3 A) and that *nckap1l* morphants display reduced *nckap1l* mRNA expression as assessed by quantitative RT-PCR (Fig. S3 B). To analyze hematopoiesis in developing zebrafish embryos, control and *nckap1l* MO-injected fish were first examined for whole-mount in situ hybridization (WISH) using probes against different hematopoietic markers, which revealed a decrease in both circulating red blood cells and neutrophils (Fig. S3, C and D; and Fig. 3 C). Following tail fin injury, we further observed fewer numbers of neutrophils at the site of injury in *nckap1l* morphants than in the embryos injected with the control MO (Fig. 3 D). Finally, and importantly, coinjection of capped WT *nckap1l* mRNA was able to rescue neutropenia in zebrafish *nckap1l* morphants (Fig. 3 E).

Although no other human cases with deficiency in *NCKAP1L* have been reported thus far, Park et al. (2008) have studied the consequences of *Nckap1l* deficiency (obtained by N-ethyl-N-nitrosourea mutagenesis) in mouse. Indeed, a homozygous premature stop codon cut short the protein at amino acid residue 445, with no protein being detectable in vivo. A number of key anomalies in the *Nckap1l^−/−^* mouse are in line with those we detected in humans and/or zebrafish. These include notably F-actin polymerization and T cell capping, which are lost, and components of the WAVE complex, which disappear in the absence of Nckap1l, suggesting, again, an essential role in T cell biology. A detailed comparison of human and mouse phenotypes further highlights other interesting findings. Interestingly, unlike typical familial HLH (characteristically linked to bicytopenia/pancytopenia), *Nckap1l^−/−^* mice, similar to our patients, display an inverse phenotype that includes leukocytosis, lymphocytosis, and monocytosis. In addition, the erythrocyte lineage shows similar subtle yet interesting defects, including a decrease in hematocrit and in mean corpuscular volume, along with reticulocytosis and anisopoikilocytosis, which collectively define a microcytic hypochromic hemolytic anemia, also reported in Rac1- and Rac2-deficient patients and mice (Kalfa et al., 2006). Of note, we observed an unexpectedly low *globin* expression in *nckap1l* knockdown fish (Fig. S3 C).

In line with the phenotype described in mice, NCKAP1L-deficient T cells show a clear impairment in F-actin polymerization consistent with a loss of WAVE complex components. This impact on cytoskeleton dynamics resembles other well-known actinopathies. Hyperinflammatory manifestations, in part fulfilling clinical HLH criteria, have been observed in a number of these diseases (Bode et al., 2015; Wegehaupt et al., 2020). HLH in NCKAP1L deficiency could be the consequence of prolonged immune stimulation due to impaired pathogen control resulting from impaired lymphocyte activation. Many of the well-known actinopathies compromise the normal signaling pathway upon activation. Impaired lymphocyte activation in actinopathies reduces the ability to fight viral infections, and HLH observed in these disorders is frequently triggered by viral, particularly EBV, infection. The fact that no viral trigger for HLH was detected in our patients does not fully exclude that also in NCKAP1L deficiency, HLH is linked to impaired pathogen control (Bucciol et al., 2020; Shoham et al., 2003). Although speculative, unchecked activation of the inflammasome could also underlie HLH in our patients. Actin cytoskeleton dynamics is crucial in inflammasome regulation (reviewed in Savic et al., 2020). Consistent with what was reported in patients with *PSTPIP1*, *CDC42*, *WDR-1*, and *ARPC1B* mutations, the high IL-1 and IL-18 levels in our NCKAP1L-deficient patients could be interpreted as an autoinflammatory signature linked to inflammasome activation, rather than the strong IFN-γ–CXCL9 signature of primary HLH.

With the near universal use of whole-exome and genome sequencing as an integral part of the diagnostic regimen of pediatric/young adult patients showing signs of a dysregulation of immunity, the number of genes linked to inborn errors of immunity (alternatively called primary immunodeficiencies) is in constant increase, with 430 officially documented as of today (Bousfiha et al., 2020). Here we report a new syndromic entity that is close, yet distinct, from familial HLH, due to recessive mutations in *NCKAP1L.* It could be best structured within so-called actinopathies (Burns et al., 2017; Janssen and Geha, 2019; Tangye et al., 2019).

## Materials and methods

### Subjects and study approval

The subjects reported in this study were members of two unrelated consanguineous families of Iranian and Saudi origins. In both families, the parents and other siblings were healthy. All family members in family 1, and all in family 2 with the exception of II.3 and II.4, were whole-exome sequenced. All subjects (and their legal guardians) gave written informed consent for genetic analyses, which was performed under the principles of the Helsinki Declaration and upon approval by the institutional review boards of the participating centers of the Tehran University Medical School (Tehran, Iran) and King Abdullah International Medical Research Center (Riyadh, Saudi Arabia).

### Exome and targeted sequencing

Genomic DNA was isolated from peripheral blood using standard protocols. Exome sequencing libraries were prepared with the Twist Human Core Exome Kit (Twist Bioscience) following the manufacturer’s recommendations. Paired-end (2 × 75 bp) sequencing was performed on a NextSeq500 sequencer (Illumina). Sequences were mapped to the hg19 reference genome using Isaac Genome Alignment Software 2.1.0 (Illumina). For each sample, median coverage was at least 60-fold, and >93% of target sequences were covered at a minimum of 10×. Sequence variants were called using the Isaac Variant Caller 2.1.0 (Illumina). Annotation was performed with the KGGSeq software package based on dbSNP build 135 (Li et al., 2012). We focused only on protein-altering variants (missense, nonsense, splice site variants, and coding indels) with alternative allele frequencies <0.005 in the 1000 Genomes Project, the Genome Aggregation Database, the Exome Aggregation Consortium, and an internal exome database including ∼700 exomes. To identify potential causal variants, we further filtered the variants based on a recessive mode of inheritance. Raw exome data (FASTQ files) are available at the National Center for Biotechnology Information’s Sequence Read Archive under the accession no. PRJNA604669.

Conventional capillary Sanger sequencing was performed to validate the genotypes of the identified candidate variants. Mutation-spanning fragments were amplified from genomic DNA using the Expand Long Template PCR System (Roche Diagnostics) according to the manufacturer’s recommendations and using the following PCR primers (5′-3′) for family 1: F1-F CAC​TCG​GAA​TAT​ATT​GAG​CAC​TGT​CCA​GAG, F1-R CTT​TGC​ATG​AGG​GAC​TCT​CTA​CAT​CAT​AGC; and family 2: F2-F AGG​CAT​TGG​TCA​TGG​TCT​TGT​G, F2-R ATT​CAA​ACC​CTC​GCC​CTT​ATC​C. After purification with the ExoProStar Kit (GE Healthcare Life Sciences), PCR products were bidirectionally sequenced using the same primers and the Big Dye Terminator Kit v3.1 (Thermo Fisher Scientific). All targeted sequencing reactions were run on an ABI PRISM 3730*xl* (Thermo Fisher Scientific).

### Whole transcriptome and proteome analyses

Each patient and three age-, sex-, and ethnically matched healthy controls (for each patient) were used for RNA-seq (*n* = 8 samples in total). Total RNA was isolated from PBMCs using the RNeasy Mini Kit (Qiagen). RNA integrity was evaluated on an Agilent Bioanalyzer 2100 using an Agilent RNA 6000 Pico Kit (Agilent Technologies). Total RNA-seq libraries were prepared with SMARTer Stranded Total RNA-Seq Kit v2 - Pico Input Mammalian (TaKaRa) according to the manufacturer’s protocol. Briefly, random primers were used for first strand synthesis, and ribosomal cDNA was cleaved by ZapR v2 in the presence of mammalian R-probes V2. Libraries were pooled and sequenced (paired-end 2 × 75 bp) on a NextSeq500 using the NextSeq 500/550 High Output Kit v2 according to the manufacturer’s instructions (Illumina). Raw RNA-seq data have been deposited in the EMBL-EBI ArrayExpress archive (accession no. E-MTAB-8524).

For each sample, quality control was performed and assessed with the NGS Core Tools FastQC. Sequence reads were mapped using STAR (Dobin et al., 2013). Unmapped reads were remapped with Bowtie2 (Langmead and Salzberg, 2012), using a very sensitive local option to optimize the alignment. The total mapped reads were finally available in Binary Alignment Map format for raw read count extraction. Read counts were determined by the HTseq-count tool of the Python package HTSeq (Anders et al., 2015) with default parameters to generate an abundance matrix. Finally, differential analyses were performed using the limma (Ritchie et al., 2015) package of the Bioconductor framework for RNA-seq data. Up- and down-regulated genes (patients vs. controls) were selected based on the adjusted P value (<0.05) and the fold change (>1.5).

For proteomic analysis, total proteins were extracted from PBMCs pellets in Laemmli buffer (10 mM Tris, pH 6.8, 1 mM EDTA, 5% β-mercaptoethanol, 5% SDS, 10% glycerol, and 1/100 anti-proteases). Protein concentrations were determined using the RC-DC protein assay (Bio-Rad Laboratories) according to the manufacturer’s instructions, and 20 µg of each protein extract was heated at 95°C for 5 min and stacked in an in-house–prepared 5% acrylamide SDS-PAGE stacking gel. Gel bands were cut, destained, reduced with 10 mM dithiothreitol, and alkylated using 55 mM iodoacetamide before overnight digestion at 37°C using modified porcine trypsin (Promega). Extracted tryptic peptides were analyzed by nano–liquid chromatography/tandem mass spectrometry (nano–LC-MS/MS) on a nano–ultra-performance liquid chromatography system (nanoAcquityUPLC, Waters Corporation) coupled to a quadrupole-Orbitrap mass spectrometer (Q-Exactive Plus, Thermo Fisher Scientific). Chromatographic separation was conducted over a 105-min linear gradient from 1 to 35% of solvent B (0.1% formic acid in acetonitrile) at a flow rate of 450 nl/min. A top 10 method was used with automatic switching between MS and MS/MS modes to acquire high-resolution MS/MS spectra. Samples were injected using a randomized injection sequence, and a solvent blank injection was performed after each sample to minimize carry-over.

Nano–LC-MS/MS data were interpreted to perform label-free extracted ion chromatogram-based differential analysis using MaxQuant (version 1.6.0.16; Tyanova et al., 2016). Peaks were assigned with the Andromeda search engine against a database containing all human entries extracted from UniProtKB-SwissProt and common contaminants (15–07-2019; 20,409 sequences, Taxonomy ID 9606). No “match between runs” was performed between the samples. The maximum false discovery rate was 1% at both peptide and protein levels with the use of a decoy strategy. Nonnormalized protein intensity values were exported and used for further statistical analysis. The complete proteomics dataset has been deposited into the ProteomeXchange Consortium via the PRIDE partner repository with the dataset identifier PXD016191 (Deutsch et al., 2017).

The raw protein intensity data for each sample were first processed using the DEP (Zhang et al., 2018) bioconductor package to assess the quality of the data. Only proteins that were observed in at least two out of the three replicates were considered. Next, the data were background-corrected and normalized by variance stabilizing transformation svn (Huber et al., 2002). Missing protein quantification values were imputed by a left-censored imputation based on random draws from a Gaussian distribution centered around a minimal value. Finally, differential analyses were performed using the limma bioconductor package (Smyth, 2004). Up- and down-regulated proteins (patients vs. controls) were selected based on the adjusted P value (<0.05) and the fold change (>1.5).

Pathway analysis of differentially expressed genes at the RNA and protein levels was completed using the Ingenuity Pathway Analysis tool (Ingenuity Systems, Qiagen). The Ingenuity knowledge base (genes only) with direct and indirect relationships was used, and only molecules and/or relationships that had been experimentally observed in rat, mouse, or human were considered. The volcano plots were generated with R using the ggplot2 library. GSEA was performed with the GSEA software from the Broad Institute (http://software.broadinstitute.org/gsea/index.jsp; Mootha et al., 2003; Subramanian et al., 2005). The gene sets from the human KEGG and reactome databases were used to compute the enrichment of differentially expressed proteins. The normalized enrichment score, nominal P value, and false discovery rate q-value were assessed using the gene_set mode with 1,000 permutations.

### Exon skipping experiments

RNA was extracted from PBMCs using the RNeasy Mini Kit (Qiagen) and reverse transcription using iScript reverse Transcription Supermix for RT-PCR (Bio-Rad Laboratories), according to the manufacturer’s instructions. For exon skipping analyses, cDNA from family 2 and an unrelated control were PCR-amplified using a forward primer hybridizing to exon 24 (5′-AGC​TGG​TGG​TGG​AAA​ACA​TG-3′) and reverse primer hybridizing to exon 27 (5′-GCC​AGC​TCA​AAG​ATA​CTC​AAG​G-3′). Amplicons were visualized on a 1.2% agarose gel using phiX174 DNA/BsuRI (HaeIII) DNA Marker 9 (Thermo Fisher Scientific).

### Western blotting

Western blot analysis was performed on 10^6^ PBMCs. Frozen cell pellets were thawed on ice before the addition of ice-cold lysis buffer (50 mM Tris-HCl, pH 8, 150 mM NaCl, 1% NP-40, 0.1% SDS, 0.1% Na deoxycholate, 1 mM dithiothreitol, 1 mM EDTA, and protease inhibitor cocktail; Roche). Cells were lysed for 1 h at 4°C while vortexing, and 15 µl of each sample (25 µg) was mixed with an equal volume of 2× loading buffer. Samples were heated for 5 min to 95°C and then centrifuged for 5 min at 16,000 *g* before loading 30 µl on a 4–20% gradient polyacrylamide gel. Electrophoresis was conducted at 200 V for 75 min before transfer to a polyvinylidene fluoride membrane using a Trans-Blot Turbo Transfer system (Bio-Rad Laboratories) according to the manufacturer’s instructions. Membranes were blocked in Tris-buffered saline, Tween 20 (0.05%), and nonfat milk (5%) for 1 h before incubation with anti-NCKAP1L polyclonal antibody (rabbit IgG; PA5-58813; Thermo Fisher Scientific; used at 1:500 dilution) and a WAVE2 (used at 1:500 dilution; WAVE-2 [D2C8] XP Rabbit mAb 3659; Cell Signaling Technologies) overnight at 4°C. Anti-GAPDH (used at 1:2,500 dilution; Millipore) was applied/incubated for 2 h at room temperature. Secondary antibodies (goat anti-rabbit and goat anti-mouse IgG [H+L]-HRP conjugates, Bio-Rad Laboratories) were used for 1 h at room temperature at 1:3,000 and 1:5,000 dilutions, respectively. Signals were revealed using the Clarity Western ECL, and detection was performed using the ChemiDoc XRS+ system (Bio-Rad Laboratories).

### Structural modeling

To build a workable structural model of NCKAP1L, we used the crystal structures of NCKAP1 from the Protein Data Bank (PDB; Berman et al., 2000) as templates. The PDB IDs were 4n78 (Chen et al., 2014) and 3p8c (Chen et al., 2010). Both PDB structures were solved within the context of the WAVE regulatory complex of *Homo sapiens*, while the NCKAP1 protein chain (chain B) only shared a root mean square deviation of 0.264 Å. Therefore, either structure could be used as a modeling template. We indeed used 4n78. The NCKAP1L protein shares a sequence identity of 59.44% with NCKAP1. In structure modeling, two proteins are expected to share the same structure fold when the sequence identity is >30%. Thus, the structure modeling of NCKAP1 should be reliable. We used SWISS-MODEL (Waterhouse et al., 2018) to build the WT NCKAP1L structure and the NCKAP1L structure without the exon26-encoded region, based on the 4n78 template, and the resulting model was superimposed to its template in the context of the WAVE complex. Mutagenesis analysis was performed using the PYMOL program (version 2. 0 Schrödinger, LLC).

### Immune phenotyping and functional assays

#### Flow cytometry analysis and cytokine measurements

Flow cytometry analysis was performed on whole blood according to previously published methods (Rosenzwajg et al., 2019). Blood subset (CD3^+^, CD4^+^, CD8^+^ T lymphocytes, CD19^+^ B lymphocytes, and CD3^−^CD56^+^ NK cells) counts (cells/microliter) were established using CYTO-STAT tetraCHROME kits with FlowCount fluorescent beads and tetra CXP software with a cytomics FC500 flow cytometer according to the manufacturer’s instructions (all from Beckman Coulter). Deep immunophenotyping of immune cell populations, using a 10-color flow cytometry panel, was performed according to standardized methods previously published using Duraclone tubes (Beckman Coulter; Pitoiset et al., 2018a, b). Cell acquisition and analysis were performed using a Gallios cytometer, and data were analyzed with Kaluza software (Beckman Coulter; Pitoiset et al., 2018a, b). Eight age-matched controls were used for each patient.

#### Early T cell activation markers

2 × 10^5^ PBMCs were incubated for 16–20 h with PHA (2.5 µg/ml, Remel) or anti-CD3/CD28 beads (two beads per cell; Invitrogen, Thermo Fisher Scientific) in 96-well flat-bottom plates. CD25 and CD69 expression was determined on CD4^+^ T cells by flow cytometry.

#### NK and CTL degranulation

NK degranulation assays were performed as described in Bryceson et al. (2012). Briefly, PBMCs were isolated and incubated in a 96-well V-bottom plate in medium at 37°C overnight. The next day, cells were either stimulated with K562 cells or incubated in medium alone in the presence of anti-CD107a-PE antibody at 37°C for 2 h. Then cells were harvested, stained with antibodies against CD3, CD56, and CD107a, and acquired on a Navios (Beckman Coulter) flow cytometer. For analysis, CD3^−^CD56^+^ NK cells were gated and assessed for surface expression of CD107a. The term ΔCD107a is the difference between the percentage of NK cells expressing surface CD107a after K562 stimulation and incubation with medium alone. CTL degranulation assays were performed with T cell blast lines or PBMCs activated for 48 h with PHA (1.25 µg/ml) and IL-2 (100 U/ml). Activated PBMCs were either stimulated with beads (three beads per cell, DynalGibco Dynabeads Human T-Activator CD3/CD28) or incubated in medium alone in the presence of anti-CD107a antibody at 37°C for 3 h. Subsequently, cells were stained with antibodies against CD3, CD8, CD56, and CD107a and acquired on a Navios (Beckman Coulter) flow cytometer. Flat-bottom 96-well plates were coated with different concentrations of plate-bound anti-CD3 (clone OKT3, eBioscience) at 37°C for 1 h. To stimulate degranulation of T cell blasts, cells were transferred into coated wells and incubated in the presence of anti-CD107a antibody at 37°C for 3 h. Subsequently, cells were stained with antibodies against CD8 and CD107a and acquired on a Navios (Beckman Coulter) flow cytometer.

#### NK cytotoxicity

NK cell cytotoxicity was analyzed in standard ^51^Cr-release assays. Frozen PBMCs were thawed and rested in medium overnight. The next day, PBMC effectors were harvested and incubated with ^51^Cr-labeled K562 target cells for 4 h at six different effector-to-target ratios in 96-well U-bottom plates. To determine the amount of ^51^Cr release into the supernatant, supernatant was transferred onto scintillant-coated 96-Well LumaPlates (PerkinElmer Life and Analytical Sciences) and measured with a TopCount scintillation counter (PerkinElmer Life and Analytical Sciences). NK-to-target ratios were calculated by multiplying the respective effector-to-target ratio with the percentage of NK cells, as determined by flow cytometry.

#### Basal F-actin staining

A total of 2 × 10^5^ PBMCs were fixed with 4% paraformaldehyde (PFA) and stained for the surface markers CD3, CD4, and CD8. Cells were subsequently permeabilized with a saponin-containing buffer and stained with 1:1,000 phalloidin-FITC (Sigma-Aldrich). FITC mean fluorescence intensity of CD4^+^ and CD8^+^ cells was determined by flow cytometry.

#### Long-term T cell blast culture

Patient and control PBMCs were stimulated with irradiated (30.9 Gy) allogeneic PBMCs as feeder cells, PHA (2.5 µg/ml, Remel) and IL-2 (1:100, cell culture supernatant from an IL-2–producing cell line), and split every 2–4 d. Every 21 d, the cells were restimulated as mentioned before. Imaging experiments were performed between days 10 and 15 after stimulation, after the second restimulation round.

#### Crawling assay

T cell blasts were incubated overnight in the medium without IL-2 and seeded on top of recombinant human ICAM-1 Fc chimera-coated slides (0.5 µg/ml, R&D) at a concentration of 5 × 10^5^ cell/ml at 37°C. After 20 min, slides were fixed with 4% PFA, washed with 0.1% BSA and 0.2% saponin-containing buffer, and stained with 1:100 phalloidin-FITC (Sigma-Aldrich). Finally, Prolong Diamond Antifade with DAPI (Invitrogen, Thermo Fisher Scientific) was used as mounting medium. Images were taken on an LSM 880 Aryscan (Zeiss) confocal microscope with a 40× water immersion objective. Criteria for quantification were the following: “leading edge” formation was judged when the cell was elongated (presumably moving). If the cell had a rounded shape, or a forward and rear end could not be recognized, the cell was scored as “not elongated.” “Normal leading edge” was assigned when a high F-actin intensity with lamellipodial morphology (evenly distributed) on the forward end of an elongated cell could be recognized. “Abnormal leading edge” was assigned when a higher intensity on the forward end could be recognized, but the morphology was not lamellipodial or evenly distributed, such as dendrites, or unevenly ruffled membrane formation.

#### IS imaging

T cell blasts were incubated overnight in medium without IL-2 and seeded on top of poly-L-lysine (0.01%, Sigma-Aldrich) and anti-human CD3-coated (10 µg/ml, UCHT1, BD PharMingen) slides at 37°C. After 6 min, slides were fixed with 4% PFA, washed with 0.1% BSA and 0.2% saponin-containing buffer, and stained with 1:100 anti-perforin Alexa Fluor 647 (BD PharMingen), 1:100 phalloidin-FITC (Sigma-Aldrich), 1:200 rabbit anti-human pericentrin (Novus), and 1:1,000 goat anti-rabbit Alexa Fluor 555 (Invitrogen, Thermo Fisher Scientific). Finally, Prolong Diamond Antifade with DAPI (Invitrogen, Thermo Fisher Scientific) was used as mounting medium. Images were taken on an LSM 880 Aryscan (Zeiss) confocal microscope with a 63× oil immersion objective. For the quantification only, cells that showed pericentrin (centrosome) staining were taken into account. “Normal IS” was assigned when the characteristic spread actin structure around the centrosome in a ring-like form could be recognized. “No IS” was assigned when the centrosome was present but F-actin was not spread in the proximity of the CD3 layer, and/or formed dendrite structures, “Distorted IS” was assigned when the centrosome was present and actin was spread in the proximity of the CD3 layer, but the form was far from “ring-like.”

### Zebrafish experiments

#### Zebrafish husbandry and genetic strains

Zebrafish were bred and maintained at 28°C as described by Nüsslein-Volhard and Dahm (2002). Staging was performed by hours postfertilization (hpf) as summarized by Warga and Kimmel (1990) and according to the Federation of European Laboratory Animal Science Associations and Swiss federal law guidelines. Experiments were performed with WT Tübingen fish.

#### Morpholino design and validation

Two *nckap1l* splice MOs to prevent premRNA splicing and a standard control MO were synthesized by Gene Tools (Gene Tools LLC): MO1, 5′-TCT​GAA​ACA​GTT​GAT​GAG​CAC​AGG​T-3′; MO2, 5′-AGA​CGC​TGA​CGG​ACT​GAC​CTT​AG-3′, and control MO, 5′-CCT​CTT​ACC​TCA​GTT​ACA​ATT​TAT​A-3′. MO1 binds to the acceptor site of exon 5, while MO2 targets the donor site of exon 6. Embryos were injected, fixed, and validated as previously described (Carapito et al., 2017). RT-PCR to validate splice modification was performed using the following primer pairs that span the respective regions: 5′-CTC​AGA​CCC​AAA​GCG​AAG​AC-3′ and 5′-GAC​CGA​ACT​CCT​CAG​ACA​GC-3′. Real-time quantitative PCR was performed as described (Konantz et al., 2016) on control and morphant fish using primer pairs for *nckap1l* (5′-GTG​ACG​GAG​GCT​GTT​CTC​TC-3′ and 5′-TCT​GAG​AGT​TTG​CGT​TGG​TG-3′) and *gapdh*. For rescue experiments, zebrafish WT *nckap1l* cDNA cloned into the pCS2^–^ expression vector (RZPD) was used. Capped mRNA was then synthesized using the AmpliCap SP6 High Yield Message Maker Kit (Cellscript). 100 pg of capped mRNA was injected into the yolk of single-cell-stage embryos together with the *nckap1l* MO to rescue the MO phenotype.

#### WISH and tail fin wounding

WISH was performed as described before (Carapito et al., 2017; Konantz et al., 2016). For wound experiments, tail fins were cut at the end of the spinal cord using a sterile scalpel blade, and the embryos were then allowed to recover at 29°C in embryo medium for 6–8 h. Embryos were then fixed overnight in 4% PFA/PBS. Immunostaining was performed to detect neutrophils at the wound site according to standard protocols using a rabbit polyclonal antibody against mpo (GTX128379; GeneTex International Corporation). Stained embryos were then mounted in 0.8% low melting agarose. Images were obtained using a Leica SP5-II-Matrix confocal microscope (Leica Microsystems AG). *mpo-* and *lyz*-positive cells from both WISH and immunostaining were semiautomatically counted using Fiji software (Schindelin et al., 2012). According to these numbers, percentages of fish with normal vs. decreased expression were defined. Normal numbers of neutrophils were defined as the mean number of cells in control MO–injected embryos.

### Online supplemental material

Fig. S1 shows additional immunophenotyping in patients and controls. Fig. S2 shows signaling pathways upon transcriptome and proteome analyses. Fig. S3 shows additional phenotypes in zebrafish knockdown. Table S1 reports other candidate variants upon exome sequencing in family 1.

## Supplementary Material

Table S1 reports other candidate variants remaining after exome data filtering in family 1.
