# Supplementary material for: NCKAP1L defects lead to a novel syndrome combining immunodeficiency, lymphoproliferation, and hyperinflammation
Source: J Exp Med. 2020 Aug 6;217(12):e20192275. doi: 10.1084/jem.20192275 (PMC7526481; doi:10.1084/jem.20192275)
Supplement: Table S1 — reports other candidate variants remaining after exome data filtering in family 1. [file JEM_20192275_TableS1.docx]

Table S1. Other candidate variants remaining after exome data filtering in family 1

|  | Recessive variants | | De novo variants | |
| --- | --- | --- | --- | --- |
| Type of variant | SNP | SNP | SNP | SNP |
| Chromosome | 1 | 7 | 11 | 12 |
| Position^a^ | 228475906 | 38543265 | 64123088 | 67703752 |
| Depth of coverage patient | 29 | 56 | 16 | 53 |
| Depth of coverage father | 30 | 67 | 15 | 57 |
| Depth of coverage mother | 22 | 74 | 13 | 36 |
| Reads for alternative allele patient | 29 | 56 | 9 | 20 |
| Reads for alternative allele father | 16 | 37 | 0 | 0 |
| Reads for alternative allele mother | 12 | 32 | 0 | 0 |
| Variant category | Missense | Missense | Missense | Missense |
| Reference/alternative allele | G/C | A/C | G/A | G/A |
| Gene | *OBSCN* | *AMPH* | *CCDC88B* | *CAND1* |
| Details of the variant | NM_001271223(116Exons):exon42:c.G11243C:p.C3748S&missense | NM_001635(21Exons):exon3:c.T190G:p.L64V&missense | NM_032251(27Exons):exon26:c.G4363A:p.A1455T&missense | NM_018448(15Exons):exon12:c.G3097A:p.A1033T&missense |
| Gene description | Obscurin, cytoskeletal calmodulin, and titin-interacting RhoGEF | Amphiphysin | Coiled-coil domain containing 88B | Cullin-associated and neddylation-dissociated 1 |
| SIFT_score | 0.649999976 | 0.029999999 | 0.07 | - |
| Polyphen2_HDIV_score | 0.001 | 0.908999979 | 0.799000025 | 1 |
| LRT_score | 0.017428 | 2.70E-05 | NaN | 0 |
| MutationTaster_score | 6.30E-05 | 0.166350007 | NaN | 0.999998987 |
| GERP++_RS | 4.110000134 | 5.920000076 | 3.039999962 | 5.210000038 |
| PhyloP | 1.319000006 | 2.266000032 | 0.871999979 | 2.604000092 |
| Minor allele frequency | 0 | 0 | 0 | 0 |
| Genotype in patient | CC | CC | GA | GA |
| Genotype in father | GC | AC | GG | GG |
| Genotype in mother | GC | AC | GG | GG |

LRT, likelihood ratio test; Polyphen2, Polymorphism Phenotyping v2; SIFT, scale-invariant feature transform; SNP, single nucleotide polymorphism.

^a^Positions refer to GRCh37 (hg19) reference genome.
